# Downstaging and laparoscopic hepatectomy plus intraoperative radiofrequency ablation for the treatment of initially unresectable multifocal hepatocellular carcinomas

**DOI:** 10.3389/fsurg.2023.1340657

**Published:** 2024-01-11

**Authors:** Jianjun Wang, Hua Luo, Long Yi, Pei Yang, Xintao Zeng

**Affiliations:** Department of Hepatobiliary Surgery, Mianyang Central Hospital, School of Medicine, University of Electronic Science and Technology of China, Mianyang, China

**Keywords:** antiangiogenic tyrosine kinase inhibitors (TKIs), anti-PD-1 antibodies, transcatheter arterial chemoembolization (TACE), hepatocellular carcinoma (HCC), radiofrequency ablation (RFA)

## Abstract

**Background:**

Using TKIs plus anti-PD-1 antibodies combined with TACE in the treatment of patients with initially unresectable multiple HCCs has a high tumour response rate, and using laparoscopic hepatectomy (LH) combined with intraoperative RFA for radical treatment of multiple HCCs after successful downstaging treatment has not been reported.

**Methods:**

Consecutive patients with multiple HCCs (≤4 lesions) who were downstaged with TKIs plus anti-PD-1 antibodies combined with TACE were analysed. Imaging examinations were performed monthly, and RECIST v1.1 criteria were used to evaluate treatment effect and resectability.

**Results:**

Forty-five consecutive patients with multiple HCCs who met the inclusion criteria received downstaging treatment with TKIs plus anti-PD-1 antibodies combined with TACE. Nine patients were successfully downstaged and met the R0 resection criteria, and 8 patients underwent surgery. Among the patients, 5 patients had BCLC stage C, and 3 patients had BCLC stage B. There were 2 lesions in 5 patients, 3 lesions in 2 patients, and 4 lesions in 1 patient. The average size of the main HCC was 8.5 cm (range: 5.4–9.1 cm), and the diameter of the remaining HCCs was 1.6 cm (range: 0.8–2.9 cm). The average time from the start of downstaging therapy to surgery was 81 days (range: 60–210 days). All 8 patients underwent LH of the main HCC, and the remaining HCCs were targeted with RFA. The mean operation time was 220 min (range 150–370 min), the average intraoperative blood loss was 260 ml (range 100–750 ml), there was no case conversion to laparotomy, and the average postoperative hospital stay was 9 days (range 7–25 days). The incidence of postoperative complications was 37.5% and there were no deaths. The average follow-up time was 18.2 months (range 6.1–22.4 months), 5 patients survived tumour-free, 2 patients had tumour recurrence, and 1 patient died.

**Conclusions:**

After successful downstaging of multiple HCCs by treatment with TKIs plus anti-PD-1 antibodies and TACE, LH combined with RFA for radical surgery is safe and feasible, and the treatment effect is satisfactory. It is worthy of clinical reference, and its long-term effects require further research for confirmation.

## Introduction

Liver cancer is a serious threat to human health, and its incidence is increasing year by year. It is estimated that by 2025, more than 1 million people will be affected by this disease every year. Hepatocellular carcinoma (HCC) is the most common type of liver cancer, accounting for approximately 90% (1). The results of the BRIDGE study showed that due to the lack of typical clinical symptoms in the early stage of HCC, 64% of HCC patients were initially diagnosed as Barcelona Liver Cancer Clinical Stage (BCLC) B or C, losing the chance of radical resection, so they received systemic therapy and/or local treatment (2). In recent years, significant progress has been made in the nonsurgical treatment of HCC. Atezolizumab plus bevacizumab, lenvatinib, and sorafenib are recommended by ASCO and other guidelines as first-line treatment drugs for advanced HCC, which brings certain survival benefits to patients with advanced HCC (3, 4). Antiangiogenic tyrosine kinase inhibitors (TKIs) combined with anti-PD-1 antibodies in the treatment of unresectable HCC can achieve an objective response rate of 36% (5). Local treatments such as transcatheter arterial chemoembolization (TACE) and radiofrequency ablation (RFA) have also achieved good results in reducing tumour volume and controlling tumour thrombi (6, 7).

Certain tumors or venous cancer thrombi in the liver can be reduced in size or even completely vanished with local, systemic, or comprehensive therapy; in the end, non-radical resectable liver cancer can become radically resectable liver cancer. Consequently, it is anticipated that downstaging treatment will enhance these patients' chances of survival. In recent years, an increasing number of studies have shown that the downstaging effect of single drug treatment is not satisfactory. TKIs plus anti-PD-1 antibodies combined with TACE or HAIC could achieve a better downstaging effect. Xiao-Dong Zhu et al. downstaged 63 BCLC stage B or C patients with TKIs plus anti-PD-1 antibodies, and 12 patients (19.0%) obtained R0 resection (8). The 1-year survival rate was 90%, and the tumour-free survival rate was 80% (8). Xiaobo Yang et al. adopted systemic therapy as well as local therapy for downstaging BCLC stage C patients with extrahepatic oligometastases, which resulted in a successful conversion rate of 31.6% (9).

Although hepatectomy after downstaging can improve the overall survival rate of patients with advanced HCC and may achieve a radical cure, it should also be recognized that drug-induced liver injury may have adverse effects on surgery. R0 resection of HCC after downstaging should not only have sufficient surgical margins to achieve radical effects but also have sufficient residual liver volume (RLV) to reduce the risk of postoperative liver failure. It is generally believed that surgical margins >1 cm and a ratio of the RLV to the standard liver volume (SLV) >40% are the two necessary conditions for the R0 resection of HCC (10, 11). BCLC stage B/C HCC patients may have multiple lesions. For small lesions located deep and close to major vessels, hepatectomy is bound to be accompanied by the loss of a large amount of functional liver parenchyma, increasing the risk of postoperative liver failure. When the diameter of HCC ≤ 3 cm, the radical effect of RFA is equivalent to that of surgical resection, and functional liver parenchyma can be preserved to the greatest extent (12). Studies have confirmed that hepatectomy combined with RFA is safe and effective in the treatment of multiple HCCs. For HCC patients with ≤4 tumour lesions and good liver function, hepatectomy combined with RFA has a better prognosis than TACE alone (13). However, there is no report on laparoscopic hepatectomy (LH) combined with intraoperative RFA for initially unresectable multiple HCCs after downstaging.

In this study, eight patients with initially unresectable multiple HCCs that were BCLC stage B/C underwent LH combined with intraoperative RFA after downstaging conversion therapy using TKIs plus anti-PD-1 antibodies combined with TACE. By analyzing the clinical data of these eight patients, our results showed that after successful downstaging of multiple HCCs by treatment with TKIs plus anti-PD-1 antibodies and TACE, LH combined with RFA for radical surgery is safe and feasible, and the short-term treatment effects is satisfactory.

## Patients and methods

### Patients

This was a retrospective analysis of consecutive patients with multiple unresectable intermediate-advanced HCC who received TKIs plus anti-PD-1 antibodies combined with TACE for downstaging. Incorporating the patient's medical history, results of enhanced CT or MRI, and assessment of alpha-fetoprotein (AFP) and protein induced by vitamin K absence-II (PIVKA-II), the diagnosis of HCC was made according to the guidelines (14). Reasons for inoperability included RLV/SLV <40%, intermediate or advanced tumour stage (BCLC B/C), poor general condition and liver function, PS score >1, and inability to tolerate surgical treatment. The basic characteristics of patients, examination results, downstaging treatment regimen, surgical conditions, postoperative recovery and other indicators were analysed. This study protocol complied with the Declaration of Helsinki and was approved by the Ethics Committee of Mianyang Central Hospital (approval number: B2020-177R). Informed consent was obtained from all patients before they received PD-1, TKI, TACE and surgical treatments.

### Systemic therapy

According to the research data and guidelines of systemic treatment of HCC, we used lenvatinib combined with camrelizumab, apatinib combined with camrelizumab, and lenvatinib combined with sintilimab. The oral dose of lenvatinib was 12 mg (body weight ≥ 60 kg) or 8 mg (body weight < 60 kg) once per day; apatinib was dosed at 750 mg once per day and camrelizumab and sintilimab at 200 mg every 3 weeks. Changes in routine blood tests, liver function, renal function, thyroid function, tumour markers, and cardiac function were monitored every 2–3 weeks. Abdominal enhanced CT or MRI and thoracic CT were performed monthly. Efficacy was assessed according to mRECIST criteria (15). Adverse reactions were assessed with Common Terminology Criteria for Adverse Events (version 4.0). When the assessment met the R0 resection criteria, systemic therapy was discontinued.

### LH and intraoperative RFA

The general condition of the patient (PS score), liver stiffness, liver function reserve [indocyanine green retention rate after 15 min (ICG R15%), Child‒Pugh classification] and imaging (CT or MRI, 3-D CT) were evaluated before the operation. After successful downstaging, the criteria for R0 resection included liver lesions ≤4, main HCC resectable, diameter of the remaining lesions ≤3 cm, Child‒Pugh graded A, ICG R15% < 20%, RLV/SLV ≥ 40%, liver lesions assessed as partial remission or stable state > 4 weeks, no systemic disease and able to tolerate LH and RFA, and no extrahepatic metastasis. Postoperative complications were classified according to the Clavien–Dindo classification (16), and liver failure was assessed according to the International Study Group of Liver Surgery (17).

### Postoperative management

If no postoperative complications occurred, the treatment was continued according to the preoperative systemic treatment plan for half a year. If there were no signs of recurrence, discontinuation of the drug was considered. Routine blood tests, liver function, tumour indexes (AFP, PIVKA-II), abdominal colour Doppler ultrasound, CT or MRI were reviewed monthly in the first 3 months after the operation and every 3–6 months after 3 months. The risk of recurrence and metastasis of multiple HCC is so high that patients treated with LH and RFA routinely receive TACE as long as they do not develop serious postoperative complications (e.g., liver failure). In our study, all patients received TACE at 1 month postoperatively. If tumour recurrence was found, RFA, reoperation or TACE or replacement of the systemic treatment plan was considered.

### Statistical analyses

Statistical analysis was performed using SPSS software (ver. 22.0, SPSS Inc., Chicago, IL). For measurement data, the median (range) was used for statistical description, and a nonparametric test (Mann–Whitney *U* test) was used for comparisons between groups. Categorical variables were described in the form of the number of cases (percentages), and comparisons between groups were performed using the *χ*^2^ test or the exact probability method. Kaplan–Meier analysis was used to determine survival at each time point. The inspection level *a* = 0.05.

## Results

### Patients and treatment

**Figure 1:**
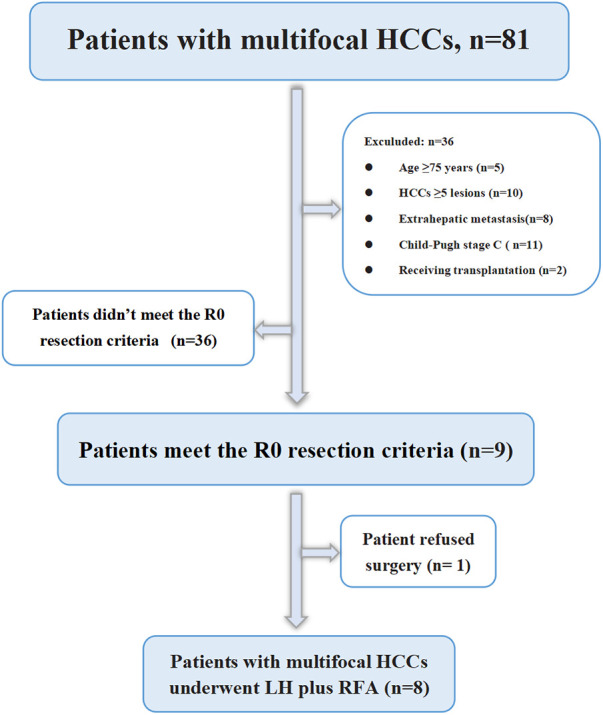
Flowchart of patient selection. HCC, hepatocellular carcinoma; LH, laparoscopic hepatectomy; RFA, radiofrequency ablation.

From January 2019 to October 2021, 81 consecutive patients with multiple HCCs were treated in our centre, of which 45 patients met the inclusion criteria and received TKIs plus anti-PD-1 antibodies combined with TACE treatment, 9 patients were downstaged successfully and met the R0 resection criteria, 1 patient was diagnosed with partial response (PR) but refused surgery and continued to maintain the original regimen; the remaining 8 patients underwent LH combined with intraoperative RFA (Figure 1). The basic data of the 45 patients who received systemic therapy and TACE are shown in Table 1. Among the 8 surgically treated patients, there were 7 males and 1 female. The median age was 53 years (range 32–65 years), the median size of the main HCC was 8.5 cm (range 5.4–9.1 cm), and the median size of the small lesions requiring RFA was 1.6 cm (range 0.8–2.9 cm). There were 2 lesions in 5 patients, 3 lesions in 2 patients, and 4 lesions in 1 patient. There were 3 cases of BCLC grade B, 5 cases of grade C, 3 cases classified under China liver cancer staging (CNLC) as IIa, 1 case of CNLC IIb, and 4 cases of CNLC IIIa. Four cases were treated with lenvatinib plus camrelizumab combined with TACE, 2 cases were treated with lenvatinib plus sintilimab combined with TACE, and 2 cases were treated with apatinib plus camrelizumab combined with TACE. Five cases of PR, 2 cases of stable disease (SD), and 1 case of complete response (CR) were evaluated by imaging (Table 2). Regarding the occurrence of adverse reactions to systemic treatment, 2 patients had fatigue less than grade 3, 1 patient had grade 3 fatigue, 1 patient had grade 3 hypertension, and no adverse reactions above grade 4 occurred. All adverse reactions of the patient were treated symptomatically and were well controlled, and the treatment regimen was not changed.

**Table 1:** Basic data of the patients with multiple HCCs.

Parameters	LH + RFA group (*n* = 8)	Nonsurgical groups (*n* = 37)	*p* values
Age, years, median (range)	53 (32–65)	51 (36–74)	0.528
Gender (male/female), *n*	7/1	31/6	0.341
BMI, (range), kg/m^2^, median (range)	20.5 (18.9–23.1)	19.2 (17.6–23.3)	0.629
Etiology (HBV/HCV/NA), *n*	8/0/0	34/2/1	0.265
Diabetes mellitus, *n* (%)	1 (12.5)	3 (8.1)	0.457
Habit of drinking, *n* (%)	2 (25.0)	9 (24.3)	0.876
BCLC stage (B/C), *n*	3/5	16/21	0.823
CNLC stage, IIa/IIb/IIIa, *n*	3/1/4	18/4/15	0.402
AFP ≥400 ng/mL, *n* (%)	3 (37.5)	15 (40.5)	0.153
Maximal lesion size, cm,median (range)	8.5 (5.4–9.1)	5.7 (3.5–12)	0.705
Number of lesions, 2/3–4, *n*	5/3	26/11	0.369
Vascular tumor thrombus, *n* (%)	3 (37.5)	11 (29.7)	0.871
Hypersplenism, *n* (%)	1 (12.5)	6 (16.2)	0.452

RFA, radiofrequency ablation; LH, laparoscopic; BMI, body mass index; HBV, hepatitis B; HCV, hepatitis C; BCLC, Barcelona clinic liver cancer; CNLC, China liver cancer; AFP, alpha-fetoprotein.

**Table 2:** Characteristics of surgical patients.

Patient No.	Maximal lesion size (cm)	Lesion number	Size of lesions by RFA (cm)	CNLC stage	BCLC stage	Vascular tumor thrombus	TKI	Anti-PD-1 antibody	Safety summary	Lesions response
1	8.5	2	2.3	IIa	B	No	Apa	Cam	Fatigue (grade 2)	PR
2	5.4	2	1.7	IIIa	C	Yes	Len	Sin	–	PR
3	6.7	4	0.9, 1.1, 2.5	IIb	C	No	Len	Cam	Fatigue (grade 2)	SD
4	7.0	2	2.6	IIa	B	No	Len	Cam	Fatigue (grade 3)	CR
5	9.1	3	2.1, 1.5	IIIa	C	Yes	Apa	Cam	–	PR
6	5.8	2	1.3	IIa	B	No	Len	Cam	–	PR
7	6.5	3	0.8, 1.5	IIIa	C	No	Len	Sin	Hypertension (grade 3)	SD
8	7.7	2	2.9	IIIa	C	Yes	Len	Cam	–	PR

CNLC, China liver cancer; BCLC, Barcelona clinic liver cancer; TKI, antiangiogenic tyrosine kinase inhibitors; Apa, apatinib; Len, lenvatinib; Sin, sintilimab; Cam, camrelizumab; PR, partial response; SD, stable disease; PD, progressive disease; CR, complete response.

### Surgery and perioperative findings

The mean time from initial treatment to surgery was 96 days (range 81–275 days), and the mean preoperative period of discontinuation of TKIs and anti-PD-1 antibodies was 8 days (range 7–10 days) and 24 days (range 21–30 days), respectively. Before surgery, all patients were classified as Child–Pugh A, RLV/SLV was 0.56 (range 0.48–0.71), ICG R15% was 9.0% (range 7.8%–15.2%), and liver stiffness was 11.2 kPa (range 9.9–18.7 kPa). The scope of LH included 1 case of SV + SVIII, 2 cases of SVI + SVII, 2 cases of SV + SVI + SVII + SVIII, and 3 cases of SII + SIII + SIV. One lesion was treated with RFA in 5 patients, 2 lesions were treated with RFA in 2 patients, and 3 lesions were treated with RFA in one patient. During the process of RFA, contrast-enhanced ultrasonography was routinely performed to help confirm the diagnosis of HCC and judge the completeness of tumour ablation. The mean operation time was 220 min (range 150–370 min), the mean intraoperative blood loss was 260 ml (range 100–750 ml), there was no case conversion to laparotomy, and the mean postoperative hospital stay was 9 days (range 7–25 days). Postoperative complications included 2 cases of liver failure, 1 case of grade A and 1 case of grade B. The grade B patient was cured by artificial liver treatment, 1 case of abdominal haemorrhage was cured by conservative treatment, and there were no deaths or unplanned reoperations. Postoperative pathological examination showed 1 case of pathological complete response (pCR), 2 cases with low differentiation, 3 cases with moderate differentiation, and 2 cases with high differentiation, as shown in Table 3.

**Table 3:** Surgical setting and pathological conditions.

Parameters	Total (*N* = 8)
Time form systemic therapy to operation, day, median (range)	96 (81–275)
TKIs withdrawal before operation, day, median (range)	8 (7–10)
Anti-PD-1 antibody withdrawal before operation, day, median (range)	24 (21–30)
RLV/SLV	0.56 (0.48–0.71)
ICG R15%	9.0 (7.8–15.2)
LSM, kPa, median (range)	11.2 (9.9–18.7)
Extent of LH, *n* (%)	
SVI + SVII	2 (25.0)
SII + SIII + SIV	3 (37.5)
SV + SVIII	1 (12.5)
SV + SVI + SVII + SVIII	2 (25.0)
Number of lesions by RFA, *n* (%)	
1	5 (62.5)
2	2 (25.0)
3	1 (12.5)
Operation time, min, median (range)	220 (150–370)
Blood loss volume, mL,median (range)	260 (100–750)
Time of portal triad clamping, min, median (range)	36 (25–58)
Patients need RBC transfusion, *n* (%)	1 (12.5)
Patients need Fresh-frozen plasma, *n* (%)	2 (25.0)
Number of patients transfer to ICU, *n* (%)	1 (12.5)
Postoperative hospital stay, day, median (range)	9 (7–25)
Postoperative complication, *n* (%)	
PHLF	2 (25.0)
Intraabdominal bleeding	1 (12.5)
Clavien-Dindo classification(II), *n*	2 (25.0)
Grade I	
Grade II	
Degree of differentiation, *n*	
Low	2 (25.0)
Moderate	3 (37.5)
High	2 (25.0)
pCR	1 (12.5)
MVI, *n* (%)	0 (0.0)

RLV, residual liver volume; SLV, standard liver volume; LSM, liver stiffness measurement; ICG R15%, indocyanine green retention rate after 15 min; RFA, radiofrequency ablation; RBC, red blood cell; ICU, intensive care unit; PHLF, Posthepatectomy liver failure; pCR, pathological complete response; MVI, Microvascular invasion.

**Figure 2:**
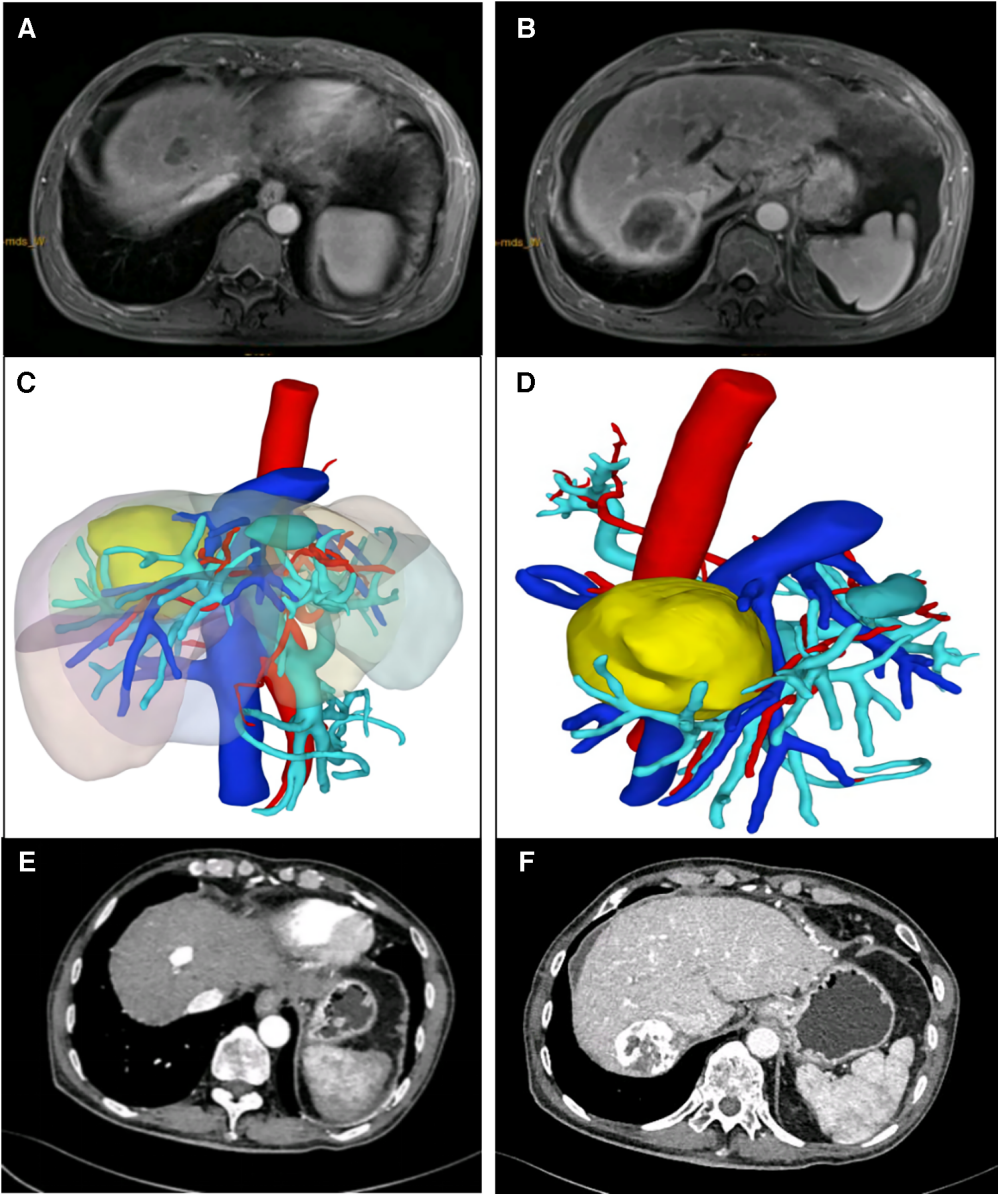
Typical case who received systemic treatment combined with TACE treatment before LH and RFA. (**A,B**) The main HCC was mainly in segment VII, and a 3 cm lesion was in segment VIII. (**C,D**) Preoperative 3-D reconstruction model of the patient, and the main HCC was close to the confluence of the right hepatic vein and the inferior vena cava. (**E,F**) CT image of the patient after 24 weeks of lenvatinib + camrelizumab combined with TACE treatment.

**Figure 3:**
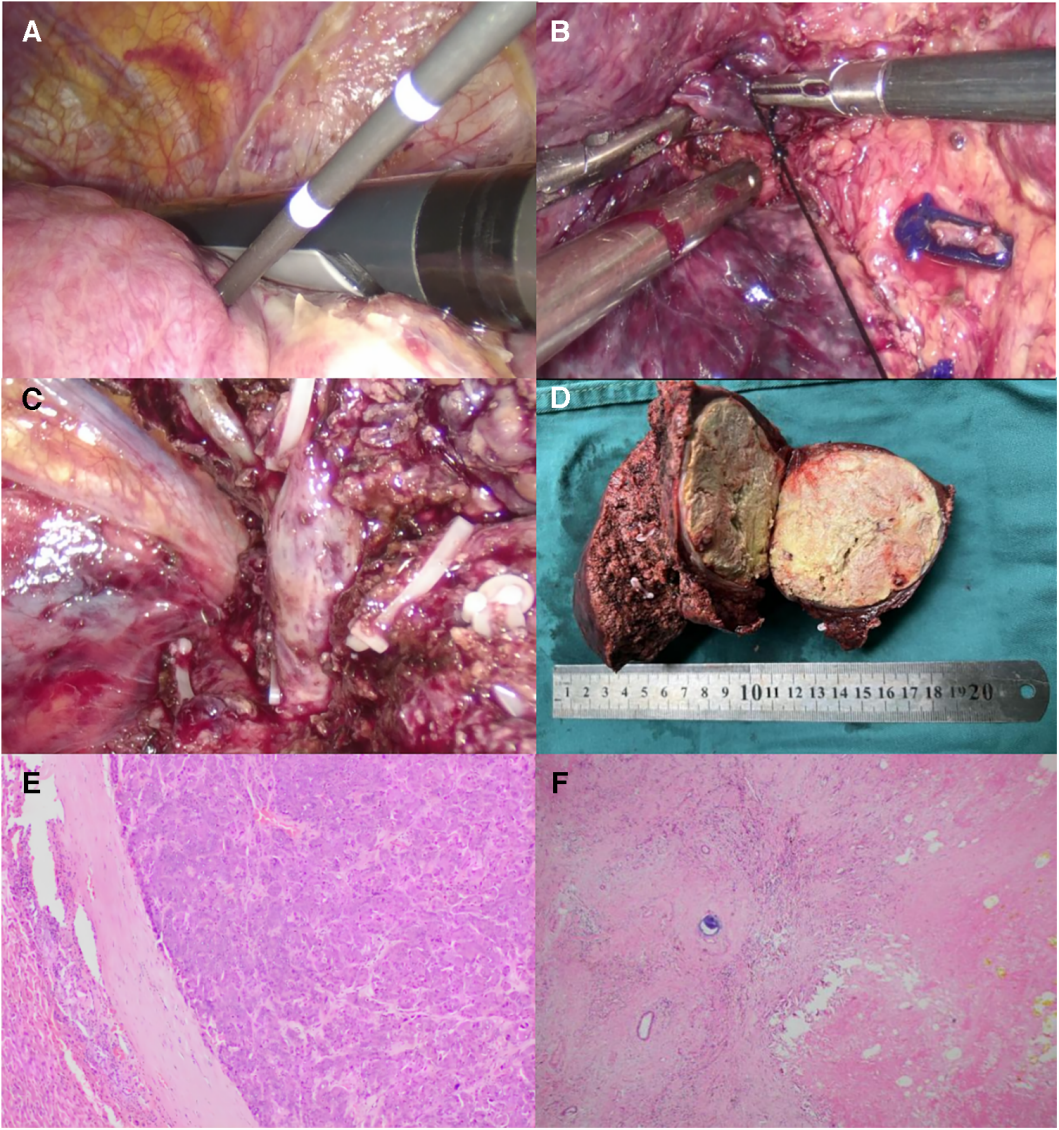
Intraoperative outcomes and postoperative pathological results. (**A**) Under intraoperative ultrasound guidance, RFA of the lesion in segment VIII was performed. (**B**) The right hepatic vein was exposed clearly. (**C**) After resection of the main HCC, the confluence of the right hepatic vein and the inferior vena cava was clearly display. (**D**) The resected main HCC of the patient, and the tumour diameter was more than 7 cm. Postoperative pathological examination (**F**) showed the patient achieved pathological complete response (pCR) compared to the needle biopsy results before systemic treatment combined with TACE (**E**).

**Figure 4:**
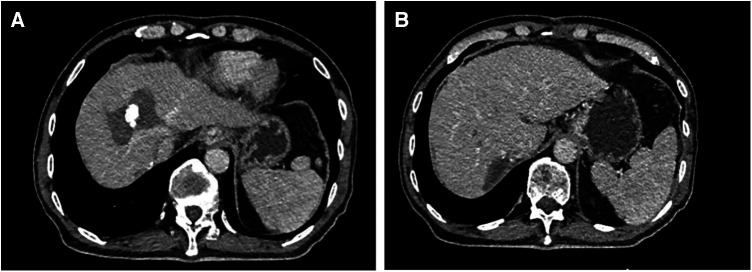
Postoperative CT images of the patient. (**A**) The CT image of the lesion in segment VIII after RFA. (**B**) The main HCC mainly in segment VII was resected after systemic treatment combined with TACE treatment.

Figures 2–4 shows one typical case. Patient 4 was diagnosed with BCLC stage B HCC. The main HCC in segment VII was close to the confluence of the right hepatic vein and the inferior vena cava, invasion of the blood vessels was considered, and there was a small lesion in segment VIII (Figures 2A–D). The biopsy results of the main HCC before systemic therapy and TACE were shown in Figure 3E. After 24 weeks of lenvatinib + camrelizumab combined with TACE treatment, the two lesions were significantly diminished (Figures 2E,F), no new nodules were found in the liver. After 2 weeks of discontinuation of systemic therapy, laparoscopic right posterior hepatectomy combined with RFA of segment VIII was performed (Figures 3A–F), postoperative pathological examination showed that no tumor cells remained in the main HCC and pCR was achieved (Figure 3F). Six months after surgery, a repeated CT scan showed no tumor recurrence or metastasis in the liver (Figures 4A,B).

### Follow-up

The follow-up cutoff time was October 1, 2021, and the median follow-up time was 18.2 months (range 6.1–22.4 months). Systemic therapy of all patients was according to the preoperative protocol. Patient 3 was found to have multiple intrahepatic metastases 6 months after the operation and received 2-line regorafenib and TACE. The patient finally died of liver failure 11 months after the operation. One recurrent liver lesion was found in patient 7 by MRI follow-up 4 months after the operation; it was confirmed to be a single nodule after TACE followed by RFA treatment, and the systemic treatment plan was changed to regorafenib. To date, no tumour recurrence has been found. The remaining 6 patients survived tumour-free. The 12-month survival rate was 7/8 (87.5%), and the tumour-free survival rate was 6/8 (75%).

## Discussion

In this study, 8 patients with initially unresectable multiple HCCs in BCLC stage B or C were treated with TKIs and anti-PD-1 antibodies combined with TACE downstaging and then underwent LH for the main HCC and intraoperative RFA for the remaining lesions. R0 resection was achieved, the 12-month postoperative survival rate was 87.5%, and the tumour-free survival rate was 75%. Systemic therapy combined with TACE downstaging followed by surgery and RFA has a good therapeutic effect on some patients with intermediate or advanced multiple HCCs.

Systemic therapy usually refers to the use of TKIs and anti-PD-1 antibodies. An increasing number of studies have shown that TKIs combined with anti-PD-1 antibodies have a higher success rate of downstaging than monotherapy (8, 9, 18). There are many combinations of TKIs and anti-PD-1 antibodies, such as lenvatinib + sintilimab and apatinib + camrelizumab. The objective response rate is 38.9%–44.0% (18–20). At present, there is a lack of comparative studies on the efficacy of different drug combinations. Therefore, in drug selection, both antitumour efficacy and safety should be taken into account. The specific mechanisms by which TKIs and anti-PD-1 antibodies enhance antitumor efficacy are still being investigated (21). Recent study supports that the efficiency of the combination downstaging therapy is the result of a series of processes, including presentation of tumor antigens, activation of T cells, infiltration of immune effector cells and identification and killing of tumor cells (22). TKIs are well-known antiangiogenetic drugs that include anti-VEGF activity. In the background of intricate cancer-immunity network, factors that promote angiogenesis, especially vascular endothelial growth factor (VEGF), can interfere with T-cell activation, infiltration and function, ultimately disrupting the anti-tumor immune response. Thus, VEGF-mediated immunosuppression provides a solid therapeutic rationale for the combination of immunotherapy with anti-angiogenic agents such as TKIs (23). Recently, some TKIs have been indicated to have anti-tumor immunomodulatory effects. For example, in mouse models and other preclinical studies, lenvatinib demonstrated an effect on the tumor microenvironment, reducing tumor-associated macrophages and increasing CD8+ T cells (24, 25). Regorafenib, a TKI with a broad range of targets including VEGF-receptor, epidermal growth factor receptor, platelet-derived growth factor receptor and fibroblast growth factor receptor, has shown anti-immunosuppressive properties and promotes anti-tumor immunity by modulating macrophages and increasing the proliferation and activation of CD8+ T cells (26). In addition, Cabozantinib has shown synergistic effects with immunotherapy in preclinical models in mice by acting on tumor-associated macrophages and reducing tumor vasculature (27). In the future, continued attention should be paid to improving the efficacy and exploring the rationality of combination therapy with TKI and anti-PD-1 antibodies. TACE is a commonly used local treatment for unresectable HCC that mainly acts by inducing tumour ischaemia and hypoxia. However, ischaemia and hypoxia can induce the release of VEGF and stimulate angiogenesis. Hypoxia can also induce changes in the tumour microenvironment and promote tumour progression. The mechanism of action of TKIs is antiangiogenesis, so systemic therapy combined with local therapy has a synergistic effect and can achieve a higher tumour remission rate and postdownstaging resection rate (28–30). At the same time, we should also fully recognize that the adverse reactions of systemic therapy and local therapy, such as immunotherapy, may lead to fatal hepatitis, enteritis, myocarditis, and pneumonia and may also lead to irreversible endocrine system damage (31). Therefore, in the process of downstaging treatment, good communication and follow-up processes should be established between doctors and patients, individualized follow-up and treatment strategies should be formulated, and multidisciplinary treatment (MDT) should be adhered to for timely diagnosis and treatment of drug-related adverse reactions.

Through systemic therapy combined with local therapy, the rate of pCR can reach 9.5%-24% (8, 32). It is uncertain whether further surgical resection is required for intermediate and advanced HCC patients who have achieved CR. A study showed that there was no significant difference in survival between hepatectomy and nonsurgical treatment for intermediate and advanced HCC after TACE achieved CR, but the study pointed out the survival benefit of surgical treatment in patients with partial remission (33). In patients with liver metastases from colon cancer, 2/3 of the lesions could reach pCR; however, more than 50% of lesions that disappear on imaging examinations will recur if they are not resected (34). For HCC patients with CR on imaging, if no surgical resection of the lesion is performed for further pathological examination, it is impossible to completely determine whether there are residual tumour cells; for HCC patients with CR, surgical resection of the lesion may result in a longer tumour-free survival time (35). Surgical treatment is not recommended for BCLC stage C HCC patients with large vessel invasion according to the BCLC guidelines, but some Asian guidelines recommend that after strict screening, some HCC patients with large vessel invasion can reach the R0 resection standard (36). Yun Huang et al. used systemic therapy combined with local therapy (TACE + RFA) for HCC patients with portal vein tumour thrombus (CNLC stage IIIa), and 33.3% of the patients achieved R0 resection after downstaging (37). Therefore, we believe that after downstaging of HCC with vascular invasion, if the patient meets the R0 resection criteria, surgical treatment should be performed.

For surgery after downstaging of HCC, preoperative 3-D CT and other imaging examinations should be fully utilized to formulate an accurate surgical plan, attempt to maintain a wide surgical margin (>1 cm) and remove the disappearing lesions to achieve R0 resection. LH has been widely applied to malignant liver tumours. In the treatment of HCC, it has the advantages of less trauma, a lower incidence of postoperative complications, and faster recovery, and the radical effect is comparable to that of laparotomy (38). LH after downstaging of HCC is extremely challenging: adhesions around the liver, hardening of the liver parenchyma, and vascular structure disorders are caused by factors such as hepatic artery embolization, chemotherapy drugs, and targeted therapy damage to the liver parenchyma, which bring great difficulties to liver dissociation, liver parenchyma dissection, and haemostasis. Intraoperative ultrasound is helpful for localization of the intrahepatic duct structure and timely correction of the surgical section. It is also helpful for the detection of residual lesions in the residual liver, and RFA can be performed at the same time (39). Current research suggests that for tumours with a diameter of ≤3 cm, the radical effect of RFA is comparable to surgical resection on tumours, and RFA is especially suitable for tumours with deep lesions that are difficult to reveal (12). LH combined with RFA, on the basis of resection of the main HCC, simultaneously treats the remaining lesions, maximizes the protection of the remaining liver function, and makes it possible to obtain a radical treatment for multiple HCCs.

This study shows that after strict screening and adequate preoperative preparation, it is safe to perform LH combined with intraoperative RFA after systemic therapy and TACE downstaging for intermediate and advanced multiple HCCs. Complications occurred in 3 of 8 patients after the operation, all of which were grade 2 according to the Clavien‒Dindo classification, including 2 cases of liver failure, 1 case of intra-abdominal haemorrhage; but there were no deaths and unplanned reoperations. Postoperative liver failure is the most common complication in hepatectomy patients after downstaging treatment, and its causes are related to factors such as insufficient RLV and drug-induced liver damage. A study has shown that a measure of preoperative liver stiffness can predict the occurrence of liver failure after hepatectomy; a liver stiffness value greater than 12 kPa indicates postoperative liver failure with a sensitivity of 52.4% and a specificity of 73.3% (40). Anti-PD-1 antibodies may lead to immune hepatitis, routine liver function tests cannot completely rule out the possibility of immune hepatitis, and needle biopsy is more meaningful (8). Similarly, TKIs may increase postoperative bleeding and affect the healing of the surgical incision. Therefore, to reduce the impact of systemic therapy on surgery, TKIs were discontinued 1–2 weeks before surgery, and anti-PD-1 antibodies were discontinued 2–4 weeks before surgery (35).

This study has the following shortcomings. First, this study was a retrospective study, and the number of cases was small. The aetiology of HCC was hepatitis B; therefore, the sample size should be increased, and a prospective randomized controlled study should be conducted to further verify the authenticity of the results. Second, the follow-up time was short, and the long-term prognosis needs further observation. Third, the sensitivities of intermediate-advanced HCC patients to downstaging are different, and the reasons need to be further studied.

In conclusion, for initially unresectable multiple HCCs treated with TKIs plus anti-PD-1 antibodies combined with TACE downstaging, after adequate preoperative preparation, LH of the main HCC combined with intraoperative RFA to treat remaining lesions gives patients with BCLC stage B/C an opportunity for radical treatment. This treatment method is safe and feasible, and the short-term tumour-free survival rate and cumulative survival rate are satisfactory. It is worthy of clinical reference, and its long-term effects require further research for confirmation.

## Data Availability

The original contributions presented in the study are included in the article/Supplementary Material, further inquiries can be directed to the corresponding author.
